# Association between serum apolipoprotein A1 and atrial fibrillation in the Chinese population: a case–control study

**DOI:** 10.1186/s12872-023-03283-y

**Published:** 2023-05-23

**Authors:** Xia Zhong, Jie Yu, Dongsheng Zhao, Jing Teng, Huachen Jiao

**Affiliations:** 1 Department of First Clinical Medical College, Shandong University of Traditional Chinese Medicine, Jinan, Shandong PR China; 2 Shandong University of Traditional Chinese Medicine, Jinan, Shandong PR China; 3 Department of Cardiology, Affiliated Hospital of Shandong University of Traditional Chinese Medicine, No. 42 Wenhua West Road, Lixia District, Jinan City, Shandong Province China

**Keywords:** Atrial fibrillation, Apolipoprotein A1, Blood lipid profiles, Inflammation, Gender

## Abstract

**Background:**

The relationship between serum apolipoprotein A1 (APOA1) and atrial fibrillation (AF) is not known. Therefore, we sought to investigate the associations between APOA1 and AF in the Chinese population.

**Methods:**

This case–control study included 950 patients with AF (29–83 years old, 50.42% male) who were hospitalized consecutively in China between January 2019 and September 2021. Controls with sinus rhythm and without AF were matched (1:1) to cases by sex and age. Pearson correlation analysis was performed to investigate the correlation between APOA1 and blood lipid profiles. Multivariate regression models were used to explore the association between APOA1 and AF. The receiver operator characteristic (ROC) curve was constructed to examine the performance of APOA1.

**Results:**

Multivariate regression analysis showed that low serum APOA1 in men and women with AF was significantly associated with AF (OR = 0.261, 95% CI: 0.162–0.422, *P* < 0.001). Pearson correlation analysis indicated that serum APOA1 was positively correlated with total cholesterol (TC) (*r* = 0.456, *p* < 0.001), low-density lipoprotein cholesterol (LDL-C) (*r* = 0.825, *p* < 0.001), high-density lipoprotein cholesterol (HDL-C) (*r* = 0.238, *p* < 0.001), and apolipoprotein B (APOB) (*r* = 0.083, *p* = 0.011). ROC curve analysis showed that APOA1 levels of 1.105 g/L and 1.205 g/L were the optimal cut-off values for predicting AF in males and females, respectively.

**Conclusion:**

Low APOA1 in male and female patients is significantly associated with AF in the Chinese population of non-statin users. APOA1 may be a potential biomarker for AF and contribute to the pathological progression of AF along with low blood lipid profiles. Potential mechanisms remain to be further explored.

## Introduction

Atrial fibrillation (AF), the most common clinically significant persistent arrhythmia, affects more than 45 million people worldwide [1–3] and is associated with an increased risk of heart failure, stroke, systemic embolism, cognitive impairment, and even death [4–8]. As a growing health threat, AF has resulted in significant morbidity, mortality, and a significant health care burden [9]. Although catheter ablation is an effective treatment for AF, the success rate of a single operation is only 60–70%, and potential complications may also exist [10–13]. There is increasing evidence that risk factor screening strategies may contribute to reducing the incidence of AF [14, 15]. Therefore, exploring available blood biomarkers associated with early pathological changes in patients with AF may help us identify potential risk factors and better understand AF pathogenesis.

Apolipoprotein A1 (APOA1), a multifunctional apolipoprotein and the key protein structural component in high-density lipoprotein (HDL) particles with well-documented cardioprotective properties, especially atheroprotective function, has been considered a significant serum biomarker for predicting multiple cardiovascular events [16, 17]. Although previous studies have reported almost no relationship between lipids and inflammatory markers [18–20], it has still been suggested that HDL-C and APOA1 had important anti-inflammatory and anti-oxidative properties [21]. In addition, current evidence suggesting the effects of dyslipidemia on AF remains controversial. Several studies have shown a negative correlation between HDL-C and AF [22–24]. One study indicated that HDL-C was positively associated with ischemic stroke in patients with AF [25], and another study suggested that there was no significant association between HDL-C and AF [26]. The relationship between blood lipid profiles and AF and its potential mechanisms deserve further investigation.

To the best of our knowledge, few systematic studies have been conducted to explore the association between serum APOA1 and AF. Therefore, we conducted a case–control study to investigate the association between serum APOA1 and sex- stratified AF and investigated the correlation between APOA1 and blood lipid profiles to help explore early potential serum biomarkers for AF.

## Methods

### Study design

**Fig. 1:**
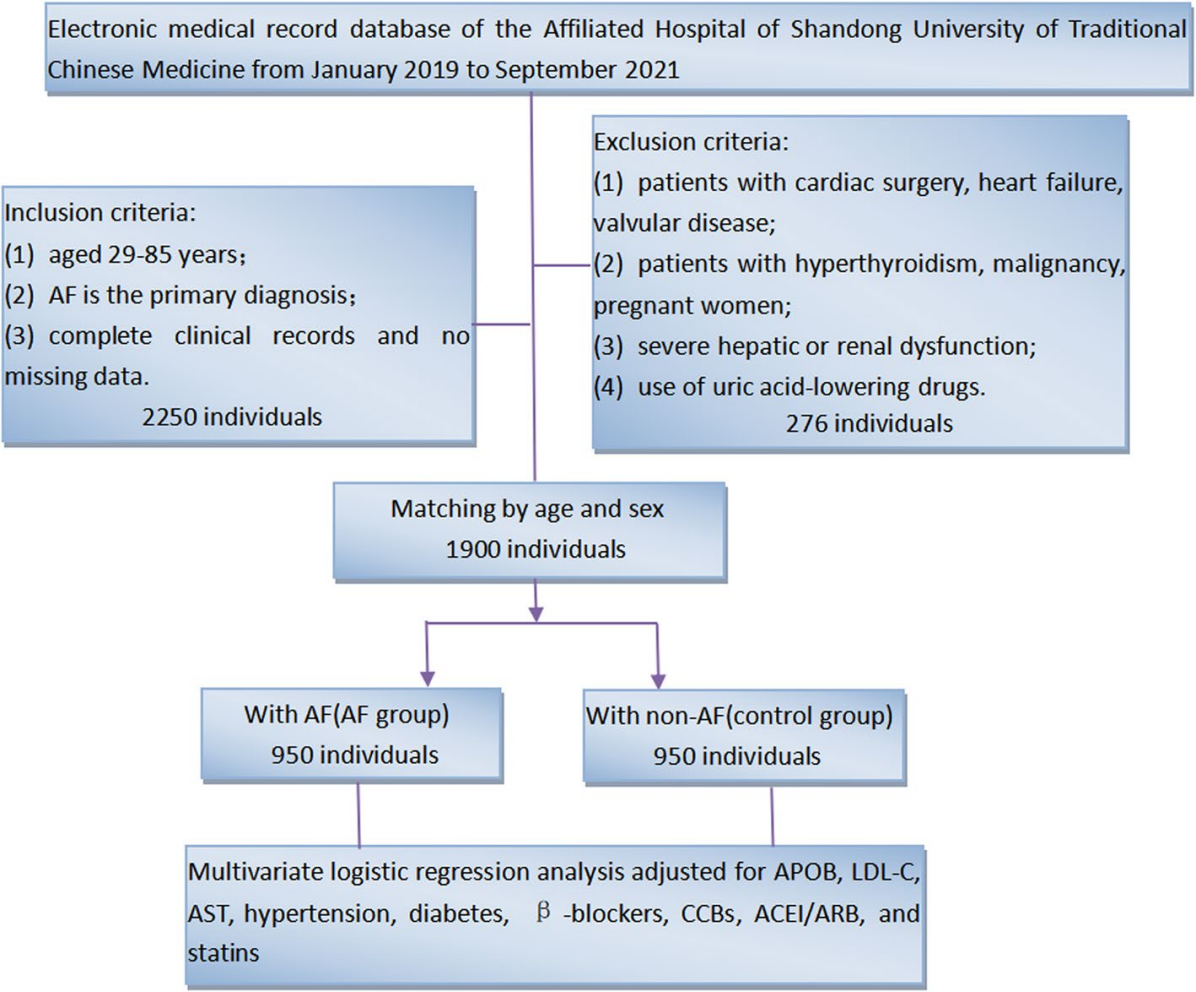
The study enrollment flowchart. Abbreviations: AF: atrial fibrillation; APOB: serum apolipoprotein B; LDL-C: low-density lipoprotein cholesterol; AST: aspartate aminotransferase

As shown in Fig. 1, we collected and analyzed clinical data from 1900 consecutive hospitalized patients (male/female: 949/951, 68.42 ± 10.87 years) from the Affiliated Hospital of Shandong University of Traditional Chinese Medicine between January 2019 and September 2021. 950 AF patients were included in the AF group, and 950 age- and sex-matched non-AF patients with sinus rhythm were used as controls. The criteria for inclusion in the AF group are: (1) 29–85 years of age; and (2) AF is the primary diagnosis; and (3) complete clinical records and no missing data. Exclusion criteria include: (1) patients with cardiac surgery, heart failure, valvular disease; or (2) patients with hyperthyroidism, malignancy, pregnant women; or (3) patients with alanine aminotransferase (ALT) > 40U/L, aspartate aminotransferase (AST) > 45U/L, or estimated glomerular filtration rate (eGFR) < 60 mL/(min 1.73m^2^); or (4) use of uric acid lowering drugs. We investigated the clinical characteristics of all participants in an electronic medical record review, including sex, age, laboratory data, AF subtypes, and complications. In addition, we stratified the information of patients with AF primarily by gender and APOB levels. We declare that this study has been reviewed by the Medical Research Ethics Committee of the Affiliated Hospital of Shandong University of Traditional Chinese Medicine (NO.20200512FA62) based on the principles of the Helsinki Declaration. The data is anonymous, so the requirement for informed consent has been waived.

### Screened variables

We selected the baseline data of all participants including sex, age, AF type, AF complication, and laboratory data including serum apolipoprotein A1(APOA1), serum apolipoprotein B (APOB), blood lipid profiles, aspartate aminotransferase(AST), alanine aminotransferase (ALT), lipoprotein (a) (Lp (a)), serum uric acid (SUA), serum creatinine (SCr), serum albumin (ALB), pre-albumin (PAB), as well as medication situation including statins, CCBs, β-blockers, ACEI/ARB. All laboratory parameters involved were measured by experts in strict compliance with hospital standards before patients were admitted for systematic treatment. APOA1 levels were determined by turbidimetric inhibition immunoassay.

### Statistical analysis

All statistical analyses were performed using SPSS software (version 26.0; SPSS Inc., Chicago, IL, USA) or GraphPad Prism software (version 9.0.0). Continuous variables were expressed as mean ± standard deviations (SD) and compared by Student t-test analysis and variance analysis (ANOVA). Categorical variables were presented as n(%) and compared by chi-square tests. Pearson correlation analyses were used to evaluate interrelationships and visualized as scatter plots. In addition, stepwise multivariate logistic regression analyses were performed to adjust for covariates, shown as odds ratios (ORs) and 95% confidence intervals (95% CIs). A *p*-value < 0.05 with two tails was considered significant.

## Results

### Baseline clinical characteristics of participants

As shown in Table 1, a total of 1900 consecutive hospitalized patients (M/F: 949/951, 68.42 ± 10.87 years) were divided into two groups, including 950 AF patients (M/F: 479/471, 68.61 ± 10.34 years) and 950 controls (M/F: 470/480, 68.23 ± 11.37 years). Compared with controls, AF patients were more likely to experience hypertension, CHD, diabetes and had a history of statins, CCBs, β-blockers, and ACEI/ARB (*p* < 0.001), as well as significantly lower levels of APOAl, APOB, TG, TC, LDL-C, HDL-C, ALB, and PAB(*P* < 0.05), significantly higher levels of AST, SCr, and SUA(*P* < 0.05), but no significant difference in serum levels of Lp(a), FBG, and ALT between the two groups (*P* > 0.05).

**Table 1:** Baseline clinical characteristics of participants

Variable	AF group (*n* = 950)	Control group (*n* = 950)	*P* value
Age, years	68.61 ± 10.34	68.23 ± 11.37	0.446
Male, n (%)	479(50.42)	470(49.47)	0.680
APOA1, g/L	1.13 ± 0.26	1.23 ± 0.25	< 0.001*
Men, g/L	1.07 ± 0.25	1.15 ± 0.24	< 0.001*
Women, g/L	1.19 ± 0.27	1.30 ± 0.23	< 0.001*
APOB, g/L	0.80 ± 0.38	0.99 ± 0.24	< 0.001*
Lp (a), mg/L	23.55 ± 26.88	22.58 ± 24.40	0.410
TC, mmol/L	4.19 ± 1.10	5.02 ± 1.10	< 0.001*
TG, mmol/L	1.24 ± 0.88	1.38 ± 1.25	0.004*
LDL-C, mmol/L	1.07 ± 0.30	1.20 ± 0.32	< 0.001*
HDL-C, mmol/L	2.50 ± 0.90	2.96 ± 0.86	< 0.001*
FBG, mmol/l	6.22 ± 2.11	6.23 ± 1.94	0.914
AST, U/L	25.30 ± 45.62	20.81 ± 10.87	0.003*
ALT, U/L	22.30 ± 27.37	20.35 ± 13.70	0.05
ALB, g/L	38.05 ± 4.64	40.09 ± 4.12	< 0.001*
PAB, g/L	19.56 ± 5.94	22.25 ± 5.52	< 0.001*
SCr, μmoI/L	78.46 ± 51.47	64.97 ± 26.65	< 0.001*
SUA, mg/dL	5.89 ± 1.75	5.11 ± 1.37	< 0.001*
Hypertension, n (%)	638(67.16)	324(34.11)	< 0.001*
CHD, n (%)	840(88.42)	240(25.26)	< 0.001*
Diabetes, n (%)	280(29.47)	157(16.53)	< 0.001*
Statins, n (%)	627(66.00)	217(22.84)	< 0.001*
CCBs, n (%)	343(36.11)	166(17.47)	< 0.001*
β-blockers, n (%)	743(78.21)	159(16.74)	< 0.001*
ACEI/ARB, n (%)	533(56.11)	139(14.63)	< 0.001*

Data were presented as mean ± SD or n(%)

*Abbreviations*: *AF* atrial fibrillation, *CHD* coronary heart disease, *APOA1* serum apolipoprotein A1, *APOB* serum apolipoprotein B, *Lp(a)* lipoprotein (a), *TC* total cholesterol, *TG* triglyceride, *LDL-C* low-density lipoprotein cholesterol, *HDL-C* high-density lipoprotein cholesterol, *AST* aspartate aminotransferase, *ALT* alanine aminotransferase, *ALB* serum albumin, *PAB* prealbumin, *SCr* serum creatinine, *SUA* serum uric acid

^*^Statistically significant (*P* < 0.05)

### Correlation between serum APOA1 and AF

We established stepwise multivariate logistic regression models to explore the correlation between serum APOA1 and AF. Covariates were determined by combining the statistical differences between the two groups as shown in Table 1 with potential influencing factors reported in previous literature. As shown in Table 2, after adjusting for APOB, LDL-C, and AST, serum APOA1 was considered to be a related factor for AF (OR = 0.232, 95% CI: 0.154–0.350, *P* < 0.001). Further, after adjusting for hypertension, diabetes, CCBs, ACEI/ARB, and statins, serum APOA1 was still an associated factor for AF (OR = 0.269, 95% CI: 0.175–0.414, *P* < 0.001). Finally, after adjusting for all confounding factors, serum APOA1 remained a significant factor for AF (OR = 0.261, 95% CI:0.162–0.422, *P* < 0.001). In addition, serum APOA1 was negatively correlated with AF in both genders (*P* < 0.05). Meanwhile, we further explored the association between APOA1 and AF in statin recipients and non-statin recipients. Interestingly, as shown in Tables 3 and 4, an independent inverse association between APOA1 and AF was found only in non-statin users (*P* < 0.05).

**Table 2:** Correlation between serum APOA1 and AF

	Total		Men		Women	
	OR 95% CI	*P* value	OR 95% CI	*P* value	OR 95% CI	*P* value
Model1	0.230 (0.160–0.332)	< 0.001*	0.241 (0.140–0.416)	< 0.001*	0.241 (0.140–0.416)	< 0.001*
Model2	0.232 (0.154–0.350)	< 0.001*	0.318 (0.176–0.575)	< 0.001*	0.123 (0.066–0.230)	< 0.001*
Model3	0.269 (0.175–0.414)	< 0.001*	0.297 (0.154–0.574)	< 0.001*	0.224 (0.121–0.415)	< 0.001*
Model4	0.261 (0.162–0.422)	< 0.001*	0.398 (0.198–0.798)	0.009*	0.120 (0.057–0.252)	< 0.001*

Model 1: crude, no adjustment

Model 2: Adjust for APOB, LDL-C, and AST

Model 3: Adjust for hypertension, diabetes, β-blockers, CCB, ACEI/ARB, and statins

Model 4: Adjust for all these factors

Abbreviations as shown in Table 1

^*^Statistically significant (*P* < 0.05)

**Table 3:** Correlation between serum APOA1 and AF with non-receiving statins

	Total		Men		Women	
	OR 95% CI	*P* value	OR 95% CI	*P* value	OR 95% CI	*P* value
Model1	0.121 (0.069–0.212)	< 0.001*	0.072 (0.029–0.176)	< 0.001*	0.115 (0.051–0.258)	< 0.001*
Model2	0.079 (0.041–0.154)	< 0.001*	0.086 (0..32–0.234)	< 0.001*	0.050 (0.019–0.132)	< 0.001*
Model3	0.172 (0.081–0.367)	< 0.001*	0.085 (0.025–0.285)	< 0.001*	0.216 (0.072–0.648)	0.006*
Model4	0.094 (0.039–0.230)	< 0.001*	0.108 (0.029–0.402)	0.001*	0.044 (0.011–0.182)	< 0.001*

Model 1: crude, no adjustment

Model 2: Adjust for APOB, LDL-C, and AST

Model 3: Adjust for hypertension, diabetes, β-blockers, CCB, and ACEI/ARB

Model 4: Adjust for all these factors

Abbreviations as shown in Table 1

^*^Statistically significant (*P* < 0.05)

**Table 4:** Correlation between serum APOA1 and AF with receiving statins

	Total		Men		Women	
	OR 95% CI	*P* value	OR 95% CI	*P* value	OR 95% CI	*P* value
Model1	0.491 (0.265–0.911)	0.024*	1.627 (0.576–4.595)	0.359	0.324 (0.140–0.749)	0.008*
Model2	0.684 (0.344–1.364)	0.281	2.877 (0.912–9.074)	0.071	0.265 (0.096-.729)	0.010*
Model3	0.666 (0.340–1.305)	0.236	2.299 (0.742–7.118)	0.149	0.415 (0.164–1.050)	0.063
Model4	0.876 (0.419–1.834)	0.726	3.900 (1.147–13.254)	0.029*	0.298 (0.096–0.929)	0.037*

Model 1: crude, no adjustment

Model 2: Adjust for APOB, LDL-C, and AST

Model 3: Adjust for hypertension, diabetes, β-blockers, CCB, and ACEI/ARB

Model 4: Adjust for all these factors

Abbreviations as shown in Table 1

^*^Statistically significant (*P* < 0.05)

### Differences in APOA1 levels between AF patients and controls by sex and age

**Fig. 2:**
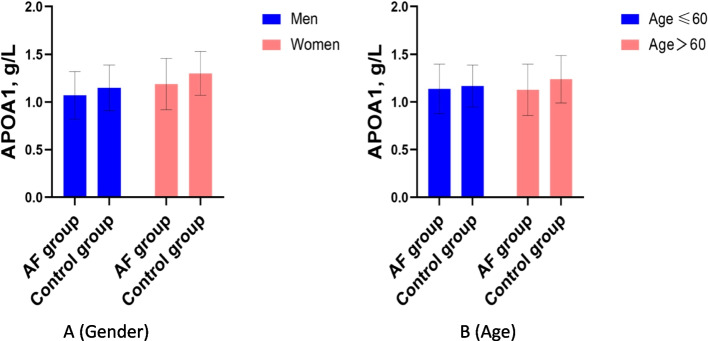
APOA1 levels in AF and control groups by sex and age. Compared with controls, APOA1 levels in AF patients were significantly lower in men( 1.07 ± 0.25 vs. 1.15 ± 0.24 g/L, *P* < 0.001; Fig. 2(**A**)) and women (1.19 ± 0.27 vs. 1.30 ± 0.23 g/L, *P* < 0.001; Fig. 2(**A**)), significantly lower in patients with age ≤ 60 years (1.14 ± 0.26 vs. 1.17 ± 0.22 g/L, *P* < 0.001; Fig. 2(**B**)) and age > 60 years (1.13 ± 0.27 vs. 1.24 ± 0.25 g/L, *P* < 0.001; Fig. 2(**B**)). Abbreviations as shown in Table 1

Figure 2 shows the difference in APOA1 levels between AF patients and controls by sex and age. Compared with controls, APOA1 levels in AF patients were significantly lower in men(1.07 ± 0.25 vs. 1.15 ± 0.24 g/L, *P* < 0.001; Fig. 2(A)) and women (1.19 ± 0.27 vs. 1.30 ± 0.23 g/L, *P* < 0.001; Fig. 2(A)), significantly lower in patients with age ≤ 60 years (1.14 ± 0.26 vs. 1.17 ± 0.22 g/L, *P* < 0.001; Fig. 2(B)) and age > 60 years (1.13 ± 0.27 vs. 1.24 ± 0.25 g/L, *P* < 0.001; Fig. 2(B)).

### Difference in APOA1 levels between AF patients and controls by type and complication of AF

**Fig. 3:**
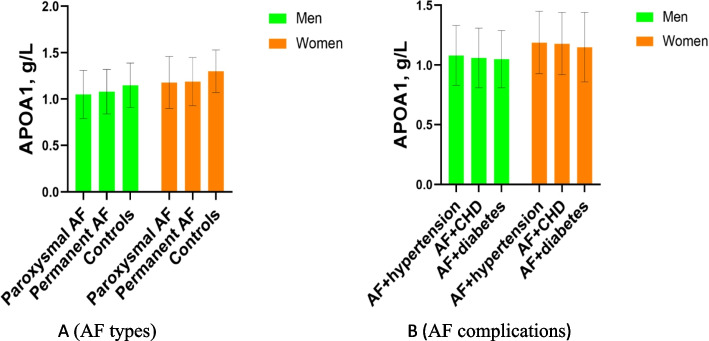
APOA1 levels in different types and complications of AF. Compared to the permanent AF group and controls, APOA1 levels in the paroxysmal AF group were significantly lower in men (1.05 ± 0. 26 vs.1.08 ± 0.24 vs. 1.15 ± 0.24 g/L, *P* < 0.001; Fig. 3(**A**)) and women ( 1.18 ± 0.28 vs. 1.19 ± 0.26 vs. 1.30 ± 0.23 g/L, *P* < 0.001; Fig. 3(**A**)). However, there was no significant difference in APOA1 levels between men and women with AF complications (*P* > 0.05; Fig. 3(**B**)). Abbreviations as shown in Table 1

Figure 3 shows the difference in APOA1 levels between AF patients and controls by type and complication of AF. Compared with the permanent AF group and controls, APOA1 levels in the paroxysmal AF group were significantly lower in the men ( 1.05 ± 0. 26 vs.1.08 ± 0.24 vs. 1.15 ± 0.24 g/L, *P* < 0.001; Fig. 3(A)) and women ( 1.18 ± 0.28 vs.1.19 ± 0.26 vs. 1.30 ± 0.23 g/L, *P* < 0.001; Fig. 3(A)). However, there was no significant difference in APOA1 levels between men and women with AF complications (*P* > 0.05; Fig. 3(B)).

### Correlation between serum APOA1 and AF-related metabolic factors

**Fig. 4:**
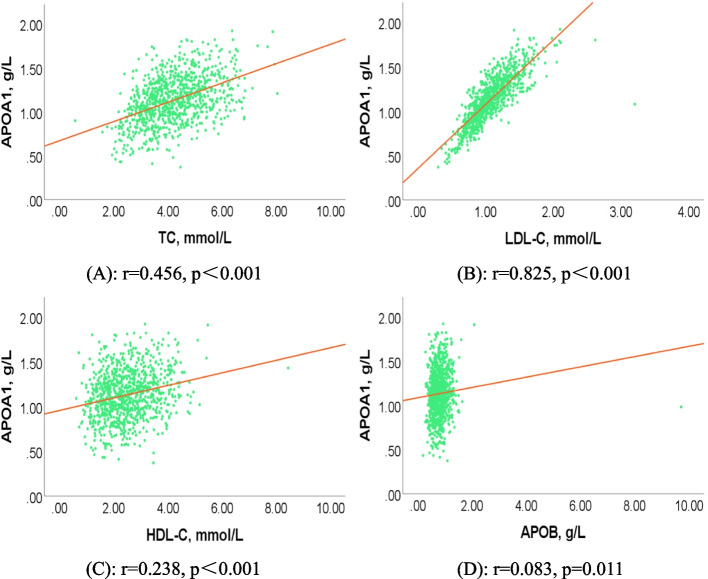
Correlation between serum APOA1 and lipid profiles in AF patients. **A** Correlation between serum APOA1 and TC in AF patients (*r* = 0.456, *p* < 0.001). **B** Correlation between serum APOA1 and LDL-C in AF patients (*r* = 0.825, *p* < 0.001). **C** Correlation between serum APOA1 and HDL-C in AF patients (*r* = 0.238, *p* < 0.001). **D** Correlation between serum APOA1 and APOB in AF patients (*r* = 0.083, *p* = 0.011). Abbreviations as shown in Table 1

Figure 4 shows the correlation between serum APOA1 and blood lipid profiles. Serum APOA1 was positively correlated with TC (*r* = 0.456, *p* < 0.001; Fig. 4(A)), LDL-C (*r* = 0.825, *p* < 0.001; Fig. 4(B)), HDL-C (*r* = 0.238, *p* < 0.001; Fig. 4(C)), and APOB (*r* = 0.083, *p* = 0.011; Fig. 4(D)).

### Subgroup analysis of serum APOA1 levels and lipid profiles in patients with paroxysmal AF

Table 5 shows the relationship between serum APOA1 levels and blood lipid profiles in patients with paroxysmal AF. These results suggested that lower serum APOA1 had lower TC, LDL-C, and HDL-C in both sexes (*P* < 0.001).

**Table 5:** The relationship between serum APOA1 levels and lipid profiles in patients with paroxysmal AF

Variable	Men(*n* = 180)				Women(*n* = 152)			
	≤ 0.97 g/L	0.97–1.16 g/L	≥ 1.16 g/L	*P* value	≤ 1.10 g/L	1.10–1.27 g/L	≥ 1.27 g/L	*P* value
Number, n	73	48	59		53	47	52	
TG, mmol/L	1.05 ± 0.54	1.12 ± 0.59	1.15 ± 0.68	0.619	1.25 ± 0.64	1.60 ± 2.33	1.20 ± 0.68	0.313
TC, mmol/L	3.47 ± 0.90	4.05 ± 0.83	4.56 ± 0.94	< 0.001*	3.69 ± 1.02	4.62 ± 1.10	5.10 ± 1.23	< 0.001*
LDL-C, mmol/L	0.77 ± 1.29	1.01 ± 0.15	1.33 ± 0.31	0.001*	0.89 ± 0.39	1.15 ± 0.21	1.41 ± 0.23	< 0.001*
HDL-C, mmol/L	2.20 ± 0.73	2.43 ± 0.74	2.59 ± 0.88	0.018*	2.15 ± 0.85	2.68 ± 0.93	3.16 ± 1.28	< 0.001*
APOB, g/L	0.73 ± 0.21	0.98 ± 1.31	0.80 ± 0.24	0.157	0.76 ± 0.22	0.83 ± 0.25	0.90 ± 0.31	0.026*

Data were presented as mean ± SD

Abbreviations as shown in Table 1

^*^Statistically significant (*P* < 0.05)

### Subgroup analysis of the relationship between serum APOA1 levels and blood lipid profiles in patients with permanent AF

Table 6 shows the relationship between serum APOA1 levels and blood lipid profiles in patients with permanent AF. The present results showed that lower serum APOA1 had lower TC, LDL-C, and HDL-C in both sexes (*P* < 0.001).

**Table 6:** The relationship between serum APOA1 levels and lipid profiles in patients with permanent AF

Variable	Men(*n* = 299)				Women(*n* = 319)			
	≤ 0.99 g/L	0.99–1.17 g/L	≥ 1.17 g/L	*P* value	≤ 1.08 g/L	1.08–1.32 g/L	≥ 1.32 g/L	*P* value
Number, n	102	94	103		108	104	107	
TG, mmol/L	1.18 ± 0.66	1.31 ± 0.83	1.28 ± 1.19	0.583	1.30 ± 0.74	1.26 ± 0.52	1.19 ± 0.45	0.378
TC, mmol/L	3.62 ± 1.03	4.05 ± 1.07	4.39 ± 1.01	< 0.001*	3.86 ± 0.99	4.38 ± 1.00	4.80 ± 0.89	< 0.001*
LDL-C, mmol/L	0.81 ± 0.13	0.99 ± 0.12	1.24 ± 0.24	< 0.001*	0.86 ± 0.19	1.12 ± 0.16	1.39 ± 0.22	< 0.001*
HDL-C, mmol/L	2.23 ± 0.81	2.43 ± 0.90	2.53 ± 0.86	0.040*	2.42 ± 0.83	2.61 ± 0.87	2.74 ± 0.81	0.020*
APOB, g/L	0.74 ± 0.24	0.77 ± 0.26	0.78 ± 0.23	0.475	0.78 ± 0.22	0.81 ± 0.24	0.82 ± 0.21	0.395

Data were presented as mean ± SD

Abbreviations as shown in Table 1

^*^Statistically significant (*P* < 0.05)

### ROC curve model for APOA1 levels predicting AF

**Fig. 5:**
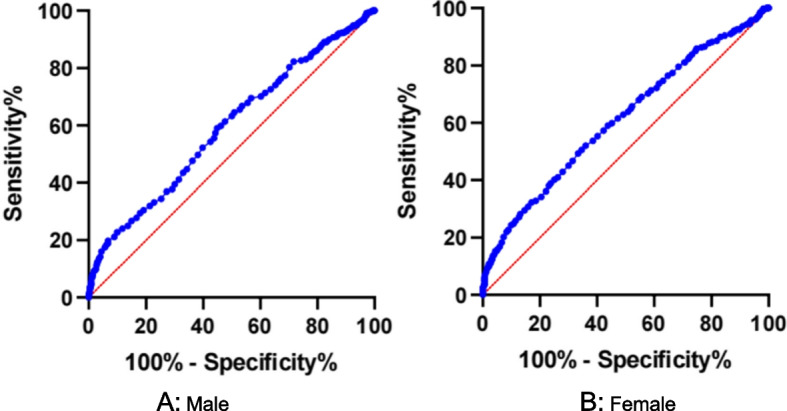
**A** ROC curve in males for APOA1 levels. The area under the ROC curve was 0.592 (0.557 to 0.628, *P* < 0.05). When the optimum cut-off value of APOA1 was 1.105 g/L, sensitivity was 0.591 and specificity was 0.551. **B** Female ROC curve for APOA1 levels. The area under the ROC curve was 0.611 (0.576 to 0.647, *P* < 0.05). When the optimum cut-off value of APOA1 was 1.205 g/L, sensitivity was 0.520 and specificity was 0.640. Abbreviations as shown in Table 1. The figure was developed by GraphPad Prism (version 9.0.0)

Figure 5 shows the ROC curve model for APOA1 levels predicting AF by sex. The ROC curve analysis suggested that APOA1 levels = 1.105 g/L were the most optimal cut-off value for predicting AF in men. Area under the ROC curve for the model was 0.592 (0.557—0.628, *P* < 0.001, Fig. 5(A)), sensitivity was 0.591, specificity was 0.551; APOA1 levels = 1.205 g/L was the best cut-off value for predicting AF in women; area under the ROC curve for the model was 0.611 (0.576—0.647, P-0.05, Fig. 5(B)), sensitivity was 0.520, specificity was 0.640.

## Discussion

This study is the first to investigate the association between serum APOA1 and AF in the Chinese population. These results indicated that low serum APOA1 in both male and female patients was associated with AF. Serum APOA1 in male patients was significantly lower than in female patients with AF. Further results showed that serum APOA1 was positively correlated with TC, LDL-C, HDL-C, and APOB. Subgroup analysis showed lower serum APOA1 in male and female patients who had lower TC, LDL-C, and HDL-C with paroxysmal AF and permanent AF. These detailed findings may contribute to our understanding of the relationship between serum APOA1 and sex in patients with AF. When the optimal cut-off value of APOA1 in males was 1.105 g/L, sensitivity was 0.591, specificity was 0.551; when the optimal cut-off value of APOA1 in females was 1.205 g/L, sensitivity was 0.520, specificity was 0.640.

As a major protein component of HDL, APOA1 has played an important role in HDL synthesis. Cholesterol acyltransferase (LCAT) is an enzyme secreted by the liver that circulates with HDL and increases HDL carrying capacity by esterifying cholesterol. Studies have shown that APOA1 not only mediated HDL synthesis but also participated in LCAT activation [17]. Clinical evidence indicated that HDL was atheroprotective, as well as having anti-inflammatory, anti-oxidative, and anti-thrombotic properties [27–29]. The oxidation of HDL mainly occurs in the inflammatory microenvironment and HDL-related paraoxonase activity may reduce systemic oxidative stress, which may be associated with a reduced risk of cardiovascular events [30, 31]. Furthermore, tryptophan oxidation of APOA1 may also contribute to promoting inflammation [32–34]. There is growing evidence that inflammation and oxidative stress play a significant role in AF pathogenesis [35–37]. In addition, oxidative modification of lipoproteins was thought to be key to atherosclerosis formation [38]. Lipid peroxidation induced damage to myocardial cells and electrophysiological changes formed the AF matrix [39–41]. From this perspective, APOA1 may maintain the pathological process of AF through pro-inflammatory and pro-oxidation chains and HDL-C.

To date, few studies have reported the association between serum APOA1 and AF. A prospective cohort study based on 35 PAF patients and 34 healthy participants showed that PAF participants had lower serum APOA1 levels [42]. Another study based on 11 women with paroxysmal lone AF and 10 women with non-AF indicated that compared with controls, there was an approximately 30% lower expression of serum APOA1 in women with AF [43]. Compared to previous studies, this study was conducted in a larger sample size and more systematically investigated the association between serum APOA1 and AF by sex, as well as blood lipid profiles. Our current results indicated that, after adjustment for confounding factors, serum APOA1 and AF were independently and negatively associated with both sexes who did not receive statins. Compared to women, APOA1 levels were significantly lower in men with AF, regardless of AF type, or AF complications. Three explanations may be suggested for this significant difference: sex hormones, arterial biochemical characteristics, and body shape [44].

In addition, we also found that serum APOA1 was correlated with blood lipid profiles. Current results showed lower blood lipid profiles in patients with AF, which was consistent with previous findings that reported significant decreases in TG and LDL-C in patients with persistent AF [45]; therefore, we speculate that our study may have included several patients with permanent AF, a phase in which patients noticed AF development and were taking lipid-lowering drugs, and thus showed lower lipid levels. Pearson correlation analysis indicated that serum APOA1 was positively correlated with TC, LDL-C, HDL-C, and APOB. Interestingly, serum APOA1 was more strongly associated with LDL-C than HDL-C, although APOA1 is a component of HDL particle, we suspect that it may be related to the stronger association between LDL-C and AF and the changes in patients' diet in the short term, this finding remains to be confirmed in future studies. Furthermore, subgroup analysis showed lower serum APOA1 in male and female patients who had lower TC, LDL-C, and HDL-C with paroxysmal AF and permanent AF. These findings suggest that the "cholesterol paradox" remains. Currently, the relationship between blood lipid profiles and AF, as well as gender differences, is still controversial. A meta-analysis of large cohort studies reported that TC, LDL-C, and HDL-C were negatively correlated with AF risk [23]. Another longitudinal study in Japan showed that low HDL-C was associated with a higher risk of new-onset AF only in women [46]. In addition, a large study from the Swedish database suggested that TC was negatively correlated with new-onset AF in men and women with hypertension [47]. Our partial results are supported by these studies, although there are some differences. Several possible reasons are worth mentioning. First, study populations are heterogeneous, due to regional differences. Second, the research and statistical methods used are different. Third, there were some confounding factors that interfered with the results. Finally, our patients were hospitalized with several complications, including hypertension, coronary heart disease, and diabetes. In addition, a relationship between low APOB and inflammation has also been established [48]. Low HDL-C is known to be associated with an increased risk of arteriosclerosis, inflammatory disorders, diabetes, and other diseases, which may lead to AF pathogenesis and risk factors [49]. Of course, the specific mechanism remains to be demonstrated in further research.

There were several potential limitations worth considering. First, this single-center case–control study cannot confirm causality. Second, we only investigated the population with paroxysmal AF and permanent AF, lacking a comparison of data for patients with persistent AF. Third, participants' status of inflammation and oxidative stress was not assessed. Fourth, some significant confounding factors may also be missing. In addition, the limited sample size was not friendly to our current results, and we gave up adjusting for several important influencing factors, such as family history, lifestyle, and exercise. However, it did provide us with a new perspective for understanding the pathology of AF. Further prospective cohort studies were encouraged, which may help further evaluate the association between serum APOA1 and AF. The effects of AF-related metabolic factors on serum APOA1 were also worth considering.

## Conclusion

In conclusion, low serum APOA1 in male and female AF patients is independently associated with AF in the Chinese population of non-statin users. Serum APOA1 levels are positively correlated with blood lipid profiles. These findings suggest that low APOA1 and blood lipid profiles may be involved in AF initiation and maintenance. Prospective cohort designs are essential to explore causal relationships and potential mechanisms.

## Data Availability

Data sets are not publicly available because they contain information that could compromise the privacy of research participants, but minimal data is available from the corresponding author upon reasonable request.
